# Risk factors for in-hospital death in 2,179 patients with acute aortic dissection

**DOI:** 10.3389/fcvm.2023.1159475

**Published:** 2023-04-25

**Authors:** Yue Yuan, Zhiyu Xia, Lei Wang, Qi Sun, Wendan Wang, Chen Chai, Tiantian Wang, Xiaowei Zhang, Long Wu, Zehai Tang

**Affiliations:** ^1^ Department of Emergency Medicine, Union Hospital, Tongji Medical College, Huazhong University of Science and Technology, Wuhan, China; ^2^ Department of Urology, Tongji Hospital, Tongji Medical College, Huazhong University of Science and Technology, Wuhan, China; ^3^ Department of Emergency Medicine, Zhongnan Hospital of Wuhan University, Wuhan, China; ^4^ Department of Cardiovascular Surgery, Union Hospital, Tongji Medical College, Huazhong University of Science and Technology, Wuhan, China

**Keywords:** acute aortic dissection, in-hospital death, the risk prediction model, admission time, surgical treatment

## Abstract

**Background:**

This study aims to investigate the risk factors for in-hospital death in patients with acute aortic dissection (AAD) and to provide a straightforward prediction model to assist clinicians in determining the outcome of AAD patients.

**Methods:**

Retrospective analysis was carried out on 2,179 patients admitted for AAD from March 5, 1999 to April 20, 2018 in Wuhan Union Hospital, China. The risk factors were investigated with univariate and multivariable logistic regression analysis.

**Results:**

The patients were divided into two groups: Group A, 953patients (43.7%) with type A AAD; Group B, 1,226 patients (56.3%) with type B AAD. The overall in-hospital mortality rate was 20.3% (194/953) and 4% (50/1,226) in Group A and B respectively. The multivariable analysis included the variables that were statistically significant predictors of in-hospital death (*P* < 0.05). In Group A, hypotension (OR = 2.01, *P* = 0.001) and liver dysfunction (OR = 12.95, *P* < 0.001) were independent risk factors. Tachycardia (OR = 6.08, *P* < 0.001) and liver dysfunction (OR = 6.36, *P* < 0.05) were independent risk factors for Group B mortality. The risk factors of Group A were assigned a score equal to their coefficients, and the score of −0.5 was the best point of the risk prediction model. Based on this analysis, we derived a predictive model to help clinicians determine the prognosis of type A AAD patients.

**Conclusions:**

This study investigate the independent factors associated with in-hospital death in patients with type A or B aortic dissection, respectively. In addition, we develop the prediction of the prognosis for type A patients and assist clinicians in choosing treatment strategies.

## Introduction

Aortic dissection (AD) is a devastating cardiovascular disease caused by a combination of a structural weakness of the aortic wall and an initiating event- dilatation of the aorta or high blood pressure tears the intima, which allows a surge of blood into the aortic wall. The blood, driven by the pulsatile pressure of the circulation, separates the layers of the arterial wall and flows into the newly created false lumen (FL), which is separated from the true lumen (TL) by the dissection membrane. This process can result in an aortic rupture in the case of adventitial disruption or in a second tear in the dissection membrane, which allows blood to reenter the TL (1–4). AD is characterized by intense cauterizing or tearing pain in the chest and back, with an onset period of less than 14 days considered acute (5). The acute aortic dissection (AAD) is a life-threatening condition associated with high mortality rate (6, 7). If AAD occurs within the ascending aorta (AA), 40% of patients die immediately (3, 8, 9). Despite continual advancements in the diagnosis and treatment, the in-hospital mortality rate of patients with acute aortic dissection remains at alarmingly levels: about 30% of patients died during hospital admission (1, 10). Therefore, to pointedly optimize treatment strategies, it is essential to identify the risk factors that increases in-hospital mortality in patients with AAD. Previous studies investigated several factors that affect the in-hospital mortality rate including complicating diseases such as hypotension, syncope, and ischaemic complications (11), as well as treatment strategy, type of AAD (6) and gender (7). However, most studies were limited by sample size or failed to provide a predictive model by using specific scores to quantify the risk factors they have investigated. Our study analysed a sufficient sample size—2,179 AAD patients over the 20 years from 1999 to 2018. Based on the analysis, we identified the risk factors affecting in-hospital mortality in patients with AAD and provided a straightforward prediction model for clinicians to have a better insight into the outcome of AAD patients.

## Materials and methods

### Patient selection

**Figure 1:**
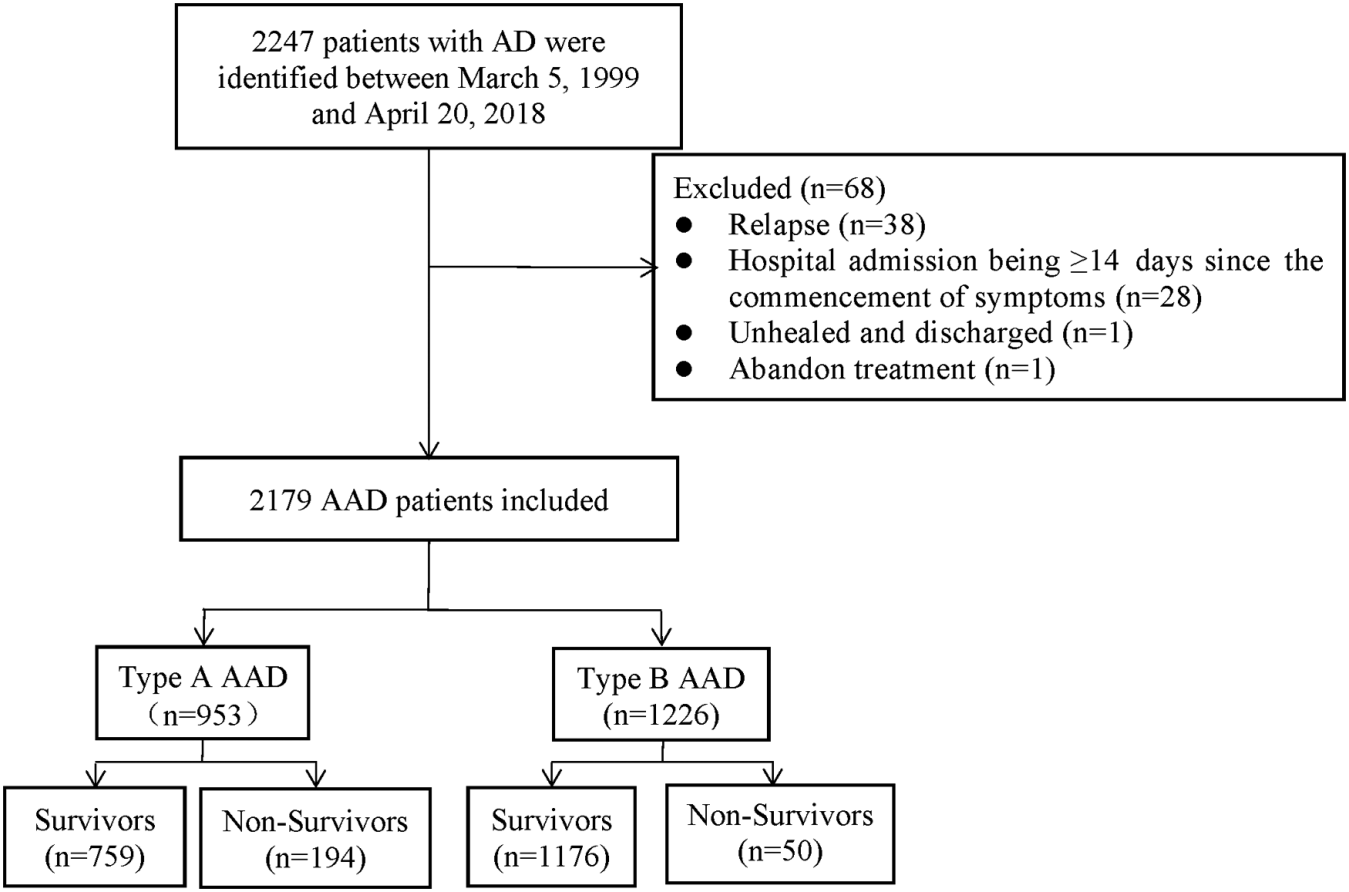
The flowchart illustrating the AAD patient selection.

A total of 2,179 AAD patients were enrolled between March 5, 1999 and April 20, 2018 in Wuhan Union Hospital of China. The patients were diagnosed by symptoms, physical examination, transthoracic echocardiography, and magnetic resonance angiography (MRA) or computed tomography angiography (CTA). Before 2001, MRA was used to determine the radiographic evidence whilst CTA was applied to clinic after 2001. Based on the Diagnosis and Treatment of Aortic Dissection published by European Society of Cardiology (ESC) and the Stanford criterion, we diagnosed and categorized AAD patients as type A or B. The exclusion criteria of the study included: (1) relapse; (2) hospital admission being ≥14 days since the commencement of symptoms; (3) unhealed and discharged; (4) abandon treatment (Figure 1).

### Data collection

The standardized data form was used to record clinical characteristics, including patient's age, sex, medical history, type of AAD, admission time, physical examination findings, laboratory examinations, method of treatment, and prognosis. Definitions of the following clinical criteria included: hypertension—blood pressure ≥140/90 mmHg; hypotension—systolic blood pressure ≤110 mmHg; tachycardia-heart rate ≥100 bpm.

### Statistical analyses

Based on Stanford classification, admitted patients were divided into two groups: patients with type A AAD, Group A; patients with type B AAD, Group B.

Descriptive statistics were used for baseline data. The normally distributed measurement data was represented as mean standard deviation (SD) and the *t*-test was performed. For the non-normally distributed measurement data, the Mann-Whitney *U* test was used and shown as Median (P_25_, P_75_). Moreover, categorical variables were expressed as proportions, and the Fisher's exact or Chi-square tests were conducted. We performed the variable significance criteria, entering variables having an unadjusted association with in-hospital death (*P* < 0.05) in the stepwise multivariable logistic regression model. Multivariate binary logistic regression analyses (backward LR method) were performed to identify the risk factor of AAD patients in-hospital death, and we conducted the Hosmer-Lemeshow test (HL test) for the final risk prediction model selection. Odds ratio (OR) and its 95% confidence interval (95%CI) were used to assess the association between the model factors and AAD patients in-hospital mortality, and *P* < 0.05 was considered that the results are significant.

Statistical analysis was done by SPSS 26.0 Software and GraphPad Prism 8.0.2 (Version 5.0; GraphPad Software). All the *P*-values are two-sided in our study.

### Development of a risk prediction model

In the multivariable analysis, the variables that were significantly associated with in-hospital death were assigned a score equal to their coefficients (*P* < 0.05). Each patient in this study had a total score, and the receiver operating characteristic (ROC) analysis was performed on each patient's score depending on their prognosis. Varying scores related to different levels of sensitivity and specificity, and we chose the appropriate point to predict in-hospital death by comparing the value of Youden index (Sensitivity + Specificity − 1).

## Results

### Patient characteristics

Among the 2,179 patients between 1999 and 2018, 759 patients (79.6%) in Group A (953 patients) survived (79.6%), while 194 (20.4%) patients died during hospitalization. The survival rate in Group B (1,226 patients) was 95.9% (1,176 patients), and the morality was 4.1% (50 patients). The mean age of Group A was 51.8 ± 11.4, with 142 (14.9%) patients older than 65 years and 48 (5.0%) patients having a medical history of CHD. About 142 (14.9%) patients had a tachycardia (heart rate ≥100 bpm) and 195 (20.5%) patients developed hypotension (systolic blood pressure ≤110 mmHg) in Group A. The mean age of Group B was 54.1 ± 11.3, with 223 (18.2%) patients older than 65 years and 39 (3.2%) patients having a medical history of CHD. About 100 (8.2%) patients had a tachycardia and 125 (10.2%) patients developed hypotension in Group B.

The study included 218 (22.9%) female patients in Group A and 210 (17.1%) female patients in Group B. In terms of onset season, in Group A, 191 (20.0%) patients were admitted to hospital with AAD attack in the spring, 158 (16.6%) in the summer, 251 (26.3%) in the autumn and 353 (37.0%) in the winter. In Group B, the data was 315 (25.7%) in the spring,189 (15.4%) in the summer, 259 (21.1%) in the autumn and 463 (37.8%) in the winter. Of the 2,179 patients, 562 (25.8%) were admitted for AAD on weekends. More than a half of the patients in Group A (510, 53.5%) underwent surgery, and 60 (6.3%) patients underwent interventional therapy. Nearly two-thirds of the Group B patients (741, 60.4%) underwent interventional therapy, and 26 (2.1%) patients underwent surgery (Tables 1-1, 1-2).

**Table 1-1:** Baseline clinical characteristics of patients with type A AAD.

Variable	Overall (*n* = 953)	Survived (*n* = 759)	Died (*n* = 194)	*P*-value
Demographics and medical history				
Age (years)	51.8 ± 11.4	51.3 ± 11.4	53.5 ± 11.5	0.014
Age ≥65 years (%)	142 (14.9)	103 (13.6)	30 (15.5)	0.497
Female, *n* (%)	218 (22.9)	178 (23.5)	40 (20.6)	0.402
Hypertension (%)	355 (37.3)	468 (61.7)	129 (66.8)	0.184
CHD (%)	48 (5.0)	31 (4.1)	17 (8.8)	0.007
Diabetes (%)	33 (3.5)	32 (4.2)	1 (0.5)	0.012
Smoking (%)	418 (44.4)	335 (44.7)	83 (42.8)	0.764
Drinking (%)	334 (35.0)	265 (34.9)	69 (35.6)	0.829
Physical examination findings				
Heart rate	83 (72, 94)	83 (72, 93)	85 (75, 97)	0.051
Tachycardia (%)	142 (14.9)	106 (14.0)	36 (18.6)	0.109
SBP (mmHg)	123 (113, 133)	124 (115, 133)	118 (106, 134)	0.003
Hypotension (%)	195 (20.5)	129 (17.0)	66 (34.0)	<0.001
DBP (mmHg)	65 (57, 72)	65 (58, 72)	63 (52, 73)	0.088
Complication				
Cardiac dysfunction (%)	172 (19.3)	135 (19.0)	37 (19.1)	0.743
Liver dysfunction (%)	39 (4.1)	9 (1.2)	30 (15.5)	<0.001
Renal dysfunction (%)	59 (6.2)	22 (2.9)	37 (19.1)	<0.001
Method of treatment				
Drug therapy (%)	383 (40.2)	256 (33.7)	127 (65.5)	<0.001
Interventional therapy (%)	60 (6.3)	58 (7.6)	2 (1.0)	<0.001
Surgical treatment (%)	510 (53.5)	445 (58.6)	65 (33.5)	<0.001
Hospitalization time				
Spring (%)	191 (20.0)	154 (20.3)	37 (19.1)	1.000
Summer (%)	158 (16.6)	127 (16.7)	31 (16.0)	1.000
Autumn (%)	251 (26.3)	193 (25.4)	58 (29.9)	1.000
Winter (%)	353 (37.0)	285 (37.5)	68 (35.1)	1.000
Weekend (%)	272 (28.5)	206 (27.1)	66 (34.0)	0.058

**Table 1-2:** Baseline clinical characteristics of patients with type B AAD.

Variable	Overall (*n* = 1,226)	Survived (*n* = 1,176)	Died (*n* = 50)	*P*-value
Demographics and medical history				
Age (years)	54.1 ± 11.3	54.0 ± 11.3	55.4 ± 11.7	0.380
Age ≥65 years (%)	223 (18.1)	213 (18.1)	10 (20.0)	0.735
Female, *n* (%)	210 (17.1)	206 (17.5)	4 (8.0)	0.080
Hypertension (%)	840 (68.5)	812 (69.0)	28 (56.0)	0.017
CHD (%)	39 (3.2)	38 (3.2)	1 (2.0)	0.980
Diabetes (%)	59 (4.8)	58 (4.9)	1 (2.0)	0.576
Smoking (%)	595 (49.1)	568 (48.7)	27 (54.0)	0.136
Drinking (%)	485 (40.1)	462 (39.7)	23 (46.0)	0.124
Physical examination findings				
Heart rate	80 (72, 88)	80 (72, 88)	89 (77, 101)	<0.001
Tachycardia (%)	100 (8.2)	84 (7.1)	16 (32.0)	<0.001
SBP (mmHg)	128 (118, 138)	128 (119, 138)	131 (114, 141)	0.985
Hypotension (%)	125 (10.2)	115 (9.8)	10 (20.0)	0.019
DBP (mmHg)	74 (67, 81)	73 (67, 88)	76 (65, 84)	0.281
Complication				
Cardiac dysfunction (%)	233 (19.0)	224 (20.0)	9 (18.0)	0.804
Liver dysfunction (%)	11 (0.9)	6 (0.5)	5 (10.0)	<0.001
Renal dysfunction (%)	56 (4.6)	47 (4.0)	9 (18.0)	<0.001
Method of treatment				<0.001
Drug therapy (%)	459 (37.4)	425 (36.1)	34 (68.0)	<0.001
Interventional therapy (%)	741 (60.4)	730 (62.1)	11 (22.0)	<0.001
Surgical treatment (%)	26 (2.1)	21 (1.8)	5 (10.0)	0.074
Hospitalization time				
Spring (%)	315 (25.7)	302 (25.7)	13 (26.0)	0.997
Summer (%)	189 (15.4)	181 (15.4)	8 (16.0)	0.954
Autumn (%)	259 (21.1)	249 (21.2)	10 (20.0)	0.872
Winter (%)	463 (37.8)	444 (37.8)	19 (38.0)	0.987
Weekend (%)	290 (23.7)	280 (23.8)	10 (20.0)	0.535

AAD, acute aortic dissection; CHD, coronary heart disease; SBP, systolic blood pressure; DBP, diastolic blood pressure; hypotension, systolic blood pressure ≤110 mmHg; tachycardia, heart rate ≥100 bpm.

### Univariate predictors of in-hospital death in AAD patients

We found the patients with type A aortic dissection had a higher in-hospital mortality rate than type B (20.4% vs. 4.1%, *P* < 0.05). Compared to those who survived, the patients who died from type A AAD were elder (*P* < 0.05) and had lower blood pressure (SBP ≤ 110 mmHg) (34.0%, *P* < 0.05). Hypotension (20.0%, *P* < 0.05) and Tachycardia (32.0%, *P* < 0.05) were significant among the patients who died from type B AAD. In terms of hospitalization time of two groups, there was no significant association between admission in different weekdays and a higher in-hospital mortality rate (*P* = 0.580, *P* = 0.535, respectively). Furthermore, medical histories of CHD was associated with higher in-hospital death rate in Group A (*P* < 0.05), while medical histories of Hypertension was in Group B (*P* < 0.05) (Tables 1-1, 1-2).

### Multivariate predictors of in-hospital death in AAD patients

In multivariate logistic regression analysis, the following factors were independently linked to higher in-hospital mortality in Group A: Hypotension (OR = 2.01, 95% CI = 1.35–2.98, *P* = 0.001) and liver dysfunction (OR = 12.95, 95% CI = 4.05–41.38, *P* < 0.001) (Table 2-1). In Group B, they were Tachycardia (OR = 6.08, 95% CI = 3.01–12.28, *P* < 0.001) and liver dysfunction (OR = 6.36, 95% CI = 1.32–30.51, *P* = 0.021) (Table 2-2). Moreover, the Interventional treatment (OR = 0.09, 95% CI = 0.02–0.36, *P* = 0.001), surgical treatment (OR = 0.21, 95% CI = 0.14–0.31, *P* < 0.001) and medical history of diabetes (OR = 0.10, 95% CI = 0.01–0.75, *P* = 0.025) were protective factors for hospitalized patients with type A AAD, whereas Interventional treatment (OR = 0.20, 95% CI = 0.10–0.41, *P* = 0.001) was protective factor for patients with type B AAD.

**Table 2-1:** Multivariate logistic regression for prediction of death with type A AAD.

Model variables	Coefficient	SE	Wald	*P*	OR (95% CI)
Interventional therapy	−2.46	0.73	11.33	0.001	0.09 (0.02–0.36)
Surgical treatment	−1.55	0.20	61.56	<0.001	0.21 (0.14–0.31)
Medical history of diabetes	−2.31	1.03	5.02	0.025	0.10 (0.01–0.75)
Hypotension	0.70	0.20	11.98	0.001	2.01 (1.35–2.98)
Liver dysfunction	2.56	0.59	18.66	<0.001	12.95 (4.05–41.38)

**Table 2-2:** Multivariate logistic regression for prediction of death with type B AAD.

Model variables	Coefficient	SE	Wald	*P*	OR (95% CI)
Interventional therapy	−1.62	0.37	19.43	<0.001	0.20 (0.10–0.41)
Tachycardia	1.81	0.36	25.35	<0.001	6.08 (3.01–12.28)
Liver dysfunction	1.85	0.80	5.34	0.021	6.36 (1.32–30.51)

### The predictive model for prognosis in-hospital patients

Based on the finding of multivariate logistic regression analysis, we assigned a score to each independent factor, and every corresponding assigned score was equal to the coefficient of each variable. The hepatic dysfunction was considered as the primary risk factor for patients in-hospital death both in Group A and B, and had the highest score (2.6, 1.9 respectively), while the hypotension and received the lowest score (1.8, 0.7 respectively) (Tables 2-1, 2-2). In group A, the other variables are assigned values: interventional therapy = −2.5, surgical treatment = −1.5, and medical history of diabetes = −2.3. Clinicians add up type A patients' scores according to the scoring criteria, and the total score of −0.5 was the best point of the risk prediction model, with sensitivity = 77% and specificity = 68% (Youden index = 45%) (Table 3). Patients with an overall score ≤−0.5 are more likely to survive, while those with higher scores were more likely to have adverse outcomes. For type B AAD, there are only three independent factors associated with hospitalized deaths, which is insufficient for developing predictive models. Hence, in order to ensure the reliability of the prediction model, we only build the model of type A AAD.

**Table 3:** The predictive model for prognosis in-hospital patients with type A AAD.

Scores	Sensitivity (%)	Specificity (%)	Youden index (%)	*P*
>−2.0	99	11	10	<0.001
>−1.0	80	60	40	<0.001
>−0.5	77	68	45	<0.001
>0	40	92	32	<0.001
>1.0	16	99	15	<0.001
>2.0	4	100	4	<0.001

## Discussion

From 1999 to 2018, Wuhan Union Hospital of China treated 2,179 patients, with 1,935 surviving and the remaining 244 dying, for a 11.2% overall in-hospital mortality. We found that despite improvements in the diagnosis and treatment of AAD over the years, there was no significant reduction in in-hospital mortality among the type A AAD patients (1999–2003: 19.0%; 2004–2008: 35.1%; 2009–2013: 16.4%; 2014–2018: 21.2%). Likewise, Pape et al. (12) discovered that no significant decrease in overall in-hospital mortality (from 12% to 14%) through a 17-year trial of 4,428 AAD patients. It is commonly acknowledged that Stanford type A is associated with an increased risk of hospitalization complications, such as cardiogenic shock, multisystem organ failure, and permanent neurological dysfunction (13–15). This may explain why patients with type A aortic dissection have a higher in-hospital mortality in our study.

In contrast to endovascular treatment and management, surgery was significantly associated with better prognosis of type A patients while interventional therapy was connected with better prognosis of type B. Tadros et al. (2) indicated that though short-term outcomes of medically managed uncomplicated TBAD are excellent, long-term outcomes are poor. Type B patients may warrant early, prophylactic endovascular intervention which were demonstrated that could improve the long-term prognosis. Ernst et al. (16) believes acute type A aortic dissections and complicated type B dissections generally require surgery, and he emphasizes the need of early surgery.

In addition, we found another protection factor independently associated with in-hospital deaths of type A AAD: Diabetes. Previous studies reported the connection between diabetes and cardiovascular diseases, Dua et al. (17) found that hyperglycemia is associated with reduced AAA diameter, increased plasma PAI-1 concentration, and reduced plasmin generation. Lederle (18) further explained that these effects may decrease aortic wall degradation directly. A meta-analysis (19) pointed out that diabetes mellitus lowers the risk of AD. Likewise, two case-control studies showed that diabetes appears to be a protective factor for the development of aortic dissection (20, 21). An observational study (22) focused on long-term risks for aortic aneurysm (AA) and aortic dissection (AD) or mortality after AA or AD hospitalization among patients with type 2 diabetes mellitus (T2DM) indicated that patients with T2DM had significantly reduced risks of AA and AD as well as reduced risk of mortality after hospitalization for AA. Identically, in our prediction model of type A AAD, the diabetes mellitus has the highest protection score, trailing only Interventional therapy, indicating that the positive effect of diabetes mellitus on patient prognosis cannot be overlooked. Hence, more prospective studies or mechanism research are in urgent need to determine a stable conclusion and to enrich related evidence, especially in the field of clarifying a potential pathophysiological association between diabetes mellitus and the prognosis of aortic dissection. Zhang et al. (11) found there was no association between a fast heart rate and in-hospital mortality in previous research involving 360 patients with aortic dissection. The number of patients enrolled in their study is restricted compared to ours. In contrary, after expanding the sample size, we discovered that tachycardia (heart rate ≥100 bpm) can potentially increases the risk of in-hospital death in type B AAD patients. The fast heart rate increases the cardiac load, leading to myocardial ischemia, while also increasing the risk of further tearing of the dissection, which may explain the preceding findings. For AAD patients, hypotension and tachycardia are two independent risk factors, which reminds our clinicians of the significance of maintaining heart rate and blood pressure within reasonable levels in clinical management. A retrospective analysis (23) pointed out that creatinine and liver malperfusion remained independent significant risk predictor to type A AAD patients. However, in our study, renal dysfunction was only significant in univariate regression (*P* < 0.001), in multivariate logistic regression analysis, the *P*-value of renal dysfunction was 0.102; whereas liver dysfunction significant in both regressions (*P* < 0.001).

Finally, a forecasting model on account of risk score was established by us to conduce to optimal treatment decision-making and prognosis assessment. The aortic dissection detection risk score is primary based on patients' anamnesis or clinical manifestation (24), and Nazerian et al. (25) indicated that integration of ADD-RS (either ADD-RS = 0 or ADD-RS ≤ 1) with DD may contribute to a more standardize rule in diagnosis of AAD. However, our model focuses more on the impact of the overall process after hospitalization, and can be used to predict patients' prognosis after receiving treatment.

Our study has some advantages. Firstly, we included 2,179 AAD patients admitted to the Wuhan Union Hospital of China from 1999 to 2018, and the sample size was sufficient. Secondly, this is the first work to analyze the effect of diabetes on the prognosis of hospitalized patients with acute aortic dissection. Thirdly, the predictive model is conducive to clinicians judging the AAD patient in-hospital outcomes. Nevertheless, some limitations should be considered when interpreting the findings. Due to the fact that this study was conducted at a single center, there is a possibility of patient selection bias. Thus, more studies are expected to demonstrate our findings. In addition, due to the large span of years of data that were analyzed in this article, several biochemical indicators cannot be counted, which could be some potential independent factors.

In summary, we found that some variables may increase the risk of in-hospital death in patients with acute aortic dissection. Furthermore, this research develops a simple and useful predictive model to assist clinicians in making more accurate treatment decisions and judging the prognosis of in-hospital patients with type A acute aortic dissection.

## Data Availability

The raw data supporting the conclusions of this article will be made available by the authors, without undue reservation.
